# Gaining new insights on the Hsp90 regulatory network

**DOI:** 10.6026/97320630016017

**Published:** 2020-01-01

**Authors:** Francesco Piva, Monia Cecati, Matteo Giulietti

**Affiliations:** 1 Department of Specialistic Clinical and Odontostomatological Sciences, Polytechnic University of Marche, Monte d'Ago, 60131, Ancona, Italy

**Keywords:** Hsp90, regulation, network

## Abstract

The heat shock protein Hsp90 is a molecular chaperon that uses ATP and interacts with various co-chaperone proteins, acting as adapters, in order to carry out the maturation of its target proteins. In physiological conditions, the heat shock proteins (HSPs) favour post-translational modification, protein folding and sub-cellular transport of their "client" proteins. In stress conditions, many misfolded proteins accumulate exposing their hydrophobic residues and these are recognized by HSPs which prevent the aggregation and favour the correct folding. In case this is no longer possible, HSPs mediate elimination of such misfolded proteins, mainly by ubiquitin–proteasome system.

## Background:

The role of HSPs in cancer is very important since cancer cells harbour mutations that cause an increased number of misfolded proteins, so high level of HSPs is required for cell survival and maintenance. In other words, molecular chaperones have an oncogenic role and have been suggested as potential therapeutic targets. Among HSPs, Hsp90 is the main target of inhibitor drugs, since its client proteins are involved in cell growth, proliferation, cell survival, and immune responses. Hsp90 undergoes a functional cycle consisting of a resting state, in which it forms a homodimer and assumes an open conformation, and an active state, in which ATP binding makes it assume an open conformation.

In humans, different members of Hsp90 exist. The cytosolic forms are: (i.) heat shock protein 90 alpha family class A member 1 (HSP90AA1) on chromosome 14 that produces the P07900-1 (732 AA) and P07900-2 (854 AA) protein isoforms; (ii.) heat shock protein 90 alpha family class A member 2 pseudogene (HSP90AA2P) on chromosome 11 that gives the Q14568-1 (343 AA) protein; iii. heat shock protein 90 alpha family class B member 1 (HSP90AB1) on chromosome 6 that yields the P08238-1 (724 AA) protein. The endoplasmic reticulum form is the heat shock protein 90 beta family member 1 (HSP90B1) on chromosome 12 of which product is the P14625-1 (803 AA) protein. The mitochondrial form is TNF receptor associated protein 1 (TRAP1) on chromosome 16 that produces the Q12931-1 (704 AA) and Q12931-2 (651 AA) protein isoforms.HSP90AB1 is constitutively expressed and HSP90AA1 is heat-inducible [1], however only HSP90AB1 seems to be essential in mammals [2,3].

**Figure 1:**
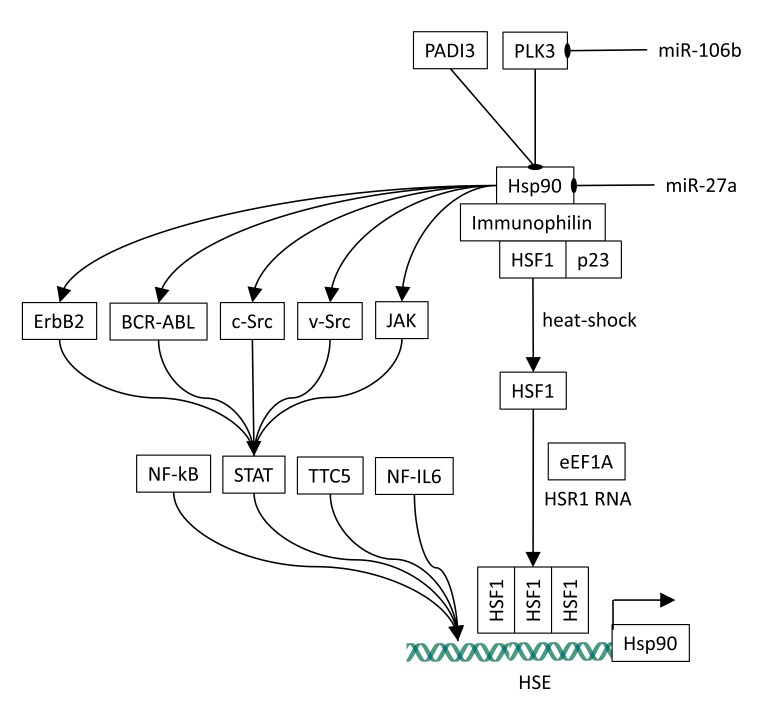
The arrows mean activation and the lines ending with a small ellipse mean inhibition. Usually, HSF1 is mainly monomeric and associated to Hsp90, p23, and immuno philin. Upon heat shock, Hsp90, p23, and immuno philin dissociate and make HSF1 free. Moreover, eE1F1 and HSR1 associate leading to the trimerization of HSF1. HSF1 trimers bind HSE sequences in promoters of heat shock-induced genes and activate their transcription. Hsp90 stabilizes proteins as ErbB2, Src, JAK and BCR-ABL that, in turn, either activate STAT by phosphorylation or enhance STAT expression. STAT promotes the transcription of Hsp90, thus forming a regulatory loop. Hsp90 is down regulated by miR-27a and by PADI3 and PLK3.

Hsp90 is usually over expressed in cancer [4], it can be secreted into the extra cellular space promoting cell motility and angiogenesis. Many efforts are being made to find selective Hsp90 inhibitors, but at the same time it is important to understand the regulatory network around Hsp90. In fact, the knowledge of the pathways allows us to understand which mechanisms increase or decrease the expression or activity of Hsp90 and can suggest new therapeutic targets. For this reason, we have performed a brief reconstruction of the pathways involving Hsp90 regulation, highlighting an interesting loop. The heat-shock response (HSR) pathway is induced by different stimuli including raised temperature, chemotherapy, bacterial or viral infection andoxidative stress. Upon these stimuli, accumulation of denatured proteins triggers the conversion of heat-shock factor 1 (HSF1) from a cytoplasmic non-DNA-binding form to a homotrimer that translocates to the nucleus it has been phosphorylated and binds heat-shock elements (HSEs) by its DNA-binding domains (DBDs) [5]. This causes the up-regulation of Hsp90 along with other chaperones and co-chaperones (Figure 1). However, also other mechanisms have been shown to regulate HSF1, for example, a ribonucleo-protein composed of the translation elongation factor eEF1A and the non-coding RNA heat-shock RNA-1 acting as a HSF1 activator [6].HSP90AB1 is up-regulated also by nuclear factor for IL-6 (NF-IL6), signal transducer and activator of transcription 3 (STAT3) and 1 (STAT1) [7] and, tetra-tri-co-peptide repeat protein 5 (TTC5) that probably acts by chromatin acetylation [8]. HSP90AA1 is up-regulated also by nuclear factor kB (NF-kB) [1]. In colorectal cancer cells, Polo-like kinase 3 (PLK3) induces Hsp90 degradation by proteasome [9]. In colon cancer, protein-arginine deiminase type-3 (PADI3) suppresses Hsp90 [10]. In esophageal squamous cell carcinoma, the over-expression of miR-27a reduced Hsp90 mRNA and protein [11]. Janus kinases (JAK1, JAK2, JAK3, and Tyrosine kinase 2 (Tyk2)), that are activator of STAT proteins, are stabilized by Hsp90, in fact Hsp90 inhibition causes the proteasome-mediated degradation of JAK1/2 [12]. Also, the activity of c-Src and v-Src, members of Src family of non-receptor tyrosine kinases that activate STAT3, depends strongly on HSP90 [12]. Among client proteins of Hsp90, the BCR-ABL fusion oncoprotein is involved in the transcriptional regulation of STAT3 and ErbB2 receptor tyrosine kinases phosphorylates STAT [12].

Current scientific knowledge does not allow us to reconstruct an accurate regulation network and this represents a limitation to the understanding of the functioning of the system, to find the stimuli that up- or down-regulate Hsp90, to know how combine chaperone inhibition, to realize the drug resistance mechanisms .In addition to the experimental approaches, the large amount of expression data present in the public databases should also be analysed more accurately to obtain new information, as was done using interesting approaches [13-16]. Overall, insights into the molecular mechanisms of Hsp90 regulatory pathway are necessary for the definition of more reliable therapeutic targets for the inhibition of Hsp90 functions in cancers.
